# Critical Overview on the Benefits and Harms of Aspirin

**DOI:** 10.3390/ph3051491

**Published:** 2010-05-14

**Authors:** Chun Shing Kwok, Yoon K. Loke

**Affiliations:** School of Medicine, University of East Anglia, Norwich, NR47TJ, UK; E-Mail: chun.kwok@uea.ac.uk (C.S.K.)

**Keywords:** aspirin, efficacy, adverse events

## Abstract

Aspirin is widely used internationally for a variety of indications, with the most prominent one being that of cardiovascular disease. However, aspirin has also been proposed as a treatment option in a diverse range of conditions such as diabetes mellitus, cancer prevention, and obstetrics. In our overview, we critically appraise the current evidence from recent systematic reviews and meta-analyses covering the benefits of aspirin across these conditions. We also look at evidence that some patients may not derive benefit due to the concept of aspirin resistance. Aspirin is also associated with the potential for significant harm, principally from haemorrhagic adverse events. We critically appraise the threat of haemorrhagic complications, and weigh up these risks against that of any potential benefit.

## 1. Introduction

The use of salicylic acid in medicine stretches back to antiquity. Early medicines containing salicylic acid were derived from willow bark and other salicylate-rich plants. These formulations were recognized for their antipyretic, analgesic and anti-inflammatory properties, but were also found to have gastrointestinal side effects. The modern form, aspirin or acetylsalicylic acid, is the acetylated version of the natural product and was developed with the aim of improving the tolerability of the drug. More recently, research into the mechanism of action of aspirin led to the discovery that it inhibited the production of prostaglandins. This has resulted in a multitude of new applications for aspirin encompassing conditions such as cardiovascular disease, pre-eclampsia, and cancer prevention. The increasing numbers of people being exposed to aspirin has also led to the awareness of the significant potential harm arising from the adverse haemorrhagic effects of aspirin (*e.g.* gastrointestinal and intracranial bleeds). Hence there is need to critically consider the evidence behind the therapeutic indications for aspirin, and decide whether the anticipated benefit outweighs the potential for harm.

In patients with high risk of vascular disease, antiplatelet therapy has been shown to reduce vascular events by about a quarter [1,2]. The benefits of aspirin treatment in secondary prevention seem to outweigh the risks, but this is much less clear for primary prevention in healthy individuals where the risk of thrombotic cardiac events is somewhat lower [3]. A number of current guidelines recommend the use of aspirin in primary prevention, but do not fully consider the risk of bleeding [4,5,6]. Aspirin is also widely used in certain subgroups of patients, e.g. patients with diabetes mellitus, and those with peripheral vascular disease, where the precise benefits have not been fully clarified.

Moreover, there has been recent concern that some patients with cardiovascular disease are aspirin resistant and do not benefit from normally recommended dose of aspirin therapy and it is unclear how to identify such patients [7,8,9,10,11,12,13]. It is not understood why patients become aspirin resistant and it may related to inadequate dosages, poor compliance, reduced ability to absorb aspirin or genetic predisposition [14,15,16,17,18,19]. The validity of platelet function testing, and its correlation with cardiovascular event, remain a hotly debated topic.

Aspirin may also have a role in obstetrics, particularly in conditions such as pre-eclampsia where vascular dysfunction in the placental beds is a potential aetiological factor. A systematic review from some years back suggests that aspirin has beneficial effects on important outcomes, with reductions in the relative risks of pre-eclampsia, preterm birth and baby death [20]. This remains a controversial area though, as early trials have shown conflicting results compared to more recent larger studies [21,22,23,24,25,26]. 

Inhibition of cyclooxygenase may have a useful role in suppression of neoplasia, as demonstrated by epidemiological studies and clinical trials that have reported reduced risk of colorectal cancer with non-steroidal anti-inflammatory agents [27,28]. There appears to be a potential association between aspirin use and a reduction in the risk of colorectal cancer [28,29,30], and colorectal adenomas [27,31,32,33]. The benefits of aspirin may also extend to other tumours such as breast cancer [34,35], and the evidence in these areas certainly merits further detailed scrutiny. Many clinical studies have evaluated the association between non-steroidal anti-inflammatory use and breast cancer and these studies have reported inconsistent results [36,37,38,39,40,41,42,43,44,45]. Two meta-analyses have pooled the data and both have shown similar a risk reduction for use of NSAIDS and aspirin compared to control. These reviews have not considered both the effect of a dose-response relationship that considers both frequency and duration of use. 

While the risks of gastrointestinal and cerebral haemorrhage with aspirin have already been studied in detail [46,47], the problems with aspirin may manifest elsewhere. The large number of patients on aspirin creates a conundrum for clinicians who are performing surgery or interventional vascular procedures on such patients, given concerns about increased risk of post-procedural bleeding from wound sites. Should aspirin be stopped prior to interventional procedures, bearing in mind the possibility of a thrombotic events? [48]. Some studies suggest that continued aspirin therapy during the operation is associated with increased risk of blood transfusion during the surgery, while other studies have suggested the contrary, including survival benefit associated with continued use of aspirin [49,50,51,52,53,54,55].

The aim of this review is to update and critically appraise the evidence from recent meta-analyses (published within the past three years) covering the common and potential applications of aspirin and evaluating the benefits and harms of treatment. 

## 2. Experimental Methods

Systematic reviews and meta-analyses that included adult patients who had aspirin therapy as a main focus were included. There was no restriction on the indication for aspirin therapy but the meta-analysis must have reported either efficacy outcomes and/or adverse events outcomes. Studies were excluded if there were more recent meta-analyses reviewing the same topic.

Recent reviews were identified by searching Medline from January 2007 until March 2010. The following terms and keywords were used [(aspirin) AND systematic[sb]]. The search was limited to meta-analyses published in English and human studies.

The studies retrieved were checked independently by two reviewers (CSK and YKL) and relevant reviews were selected according to the stated inclusion and exclusion criteria. Disagreements were resolved by consensus.

Data was extracted by CSK and checked by YKL. The characteristics of the participants included in each meta-analysis were extracted, including the total number of patients, mean age, percentage of females, the types of studies included, the main results and the validity of the primary studies based on the reviewer’s assessment.

Where available, number needed to treat for benefit (NNTB) and harm (NNTH) were extracted directly from the meta-analyses of controlled trials. If not, we extracted the event rates (%) for the pooled control and aspirin arms in order to illustrate the absolute risk reduction/increases with aspirin. The NNTB and NNTH with 95% confidence intervals (95% CI) for selected key outcomes was then calculated by applying the relative risk to the control event rate using the Visual Rx website. (http://www.nntonline.net/visualrx/) 

## 3. Results and Discussion

**Figure 1:**
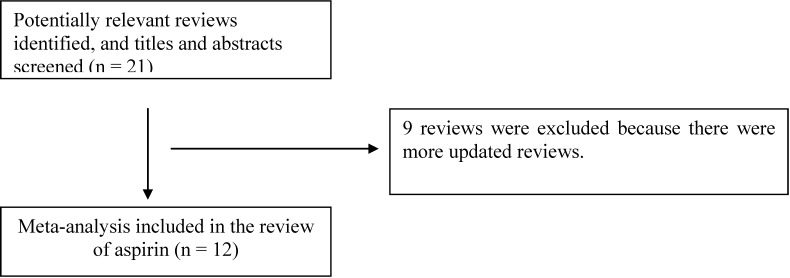
Flow diagram of selection of meta-analysis included in review.

We identified 21 potentially relevant meta-analyses articles eligible for our overview. Of these, nine were excluded because there were more recent reviews with additional studies. Of the 12 relevant analyses, the key ones with quantitative data on absolute and relative risk estimates are shown in Table 1. Study selection is shown in Figure 1. The characteristics of the trials included in the meta-analyses of this review are shown in Table 1, and the NNT for key outcomes are summarized in Table 2.

**Table 1:** Characteristics of key meta-analyses in current review.

Study	Trials and design	Participants	Outcomes	Findings	Quality
Alghamdi 2007 [62]	10 studies; 5 trials, 5 cohort studies	1,748 participants; 913 aspirin group, 835 control group	Risk of bleeding in coronary artery bypass graft patients	Aspirin use was associated with increase in blood loss, red cell and fresh frozen plasma transfusion, but not platelets or reexploration.	Results were limited because of heterogeneity and poor methodological quality
Askie 2007 [58]	31 trials	32,217 women and 32,819 babies	Risk of pre-eclampsia and pregnancy outcomes	Aspirin associated with reduced risk of pre-eclampsia, preterm delivery <34 weeks but no effect on maternal or fetal outcomes.	Trial quality was not discussed
ATC 2009 [3]	22 trials, 6 primary prevention, 16 secondary prevention	95,000 participants in primary prevention, 17,000 participants in secondary prevention trials	Risk of cardiovascular events, stroke, coronary events and death	Primary prevention with aspirin therapy results showed significant reduction in serious vascular events, non-fatal MI but not stroke or vascular mortality. Secondary prevention with aspirin significantly reduced serious vascular events, stroke and coronary events.	Trial quality was not discussed
				Significant increase in major extracranial bleeds.	
Berger 2009 [57]	18 trials	5,269 participants; 2,823 aspirin group, 2,446 control group	Risk of cardiovascular events, stroke, coronary events, death and bleeding	Aspirin therapy significantly reduced incidence of non-fatal stroke but not all-cause mortality or MI.	Quality was assessed in 12 trials, high quality (Jadad 4–5) in 6 trials and low quality (Jadad 1–3) in 6 trials
Bujold 2009 [59]	9 trials	1,317 women	Risk of pre-eclampsia and pregnancy outcomes	Aspirin therapy before <16 weeks was associated with reduced pre-eclampsia, severe preeclampsia and gestational hypertension.	Mixed quality of trials as 5/9 were double-blind, 6/9 used ITT and most had <6% loss to follow up
Cole 2009 [60]	4 trials	2,967 participants; 1,289 control, 1,678 aspirin	Risk of adenomas and adverse events	Aspirin therapy significantly reduced both adenomas and advanced lesions compared to placebo.	One trial was small and had many drop outs and there was no formal quality assessment
De Berardis 2009 [56]	6 trials	10117 participants	Risk of cardiovascular events, death and adverse events	Aspirin therapy in diabetes patient was associated with no significant reduction in cardiovascular events, cardiovascular mortality or overall mortality.	Quality was suboptimal in some studies, 3/6 had adequate allocation concealment, all studies were adequately blinded and 5/6 used ITT principles
Kraspoulos 2008 [13]	20 studies; 1 trial, 19 cohort studies	2,930 participants	Risk of cardiovascular events, stroke, coronary events, death and vascular interventions	Among aspirin resistant patient there is significantly increased risk of cardiovascular event, death and acute coronary syndrome.	Quality of trials was high in 17/20 trials and remaining trials lacked information on quality
Lewis 2007 [63]	6 cohorts studies, 4 included aspirin	1,373 participants	Risk of complications in surgery	Aspirin therapy was associated with statistically significant increase in complications.	All studies had limitations and potential biases due to observational designs
Mangiapane 2008 [61]	10 cohort studies	236,655 participants	Risk of breast cancer	Aspirin therapy was associated with statistically significant reduction in breast cancer.	Quality was not discussed

MI = myocardial infarction; ITT = intention to treat analysis

**Table 2:** Number Needed to Treat for Key Outcomes in Meta-Analyses of Aspirin *versus* Control.

Application of aspirin	Risk of event (95% confidence interval)	Treated event rate	Control event rate	Number needed to treat
**Primary and secondary prevention of cardiovascular disease**				
Primary prevention of non-fatal MI	RR 0.77 (0.67–0.89)	0.18% per year	0.23% per year	1891 (NNTB 1318– 3953) per year
Secondary prevention of non-fatal MI	RR 0.69 (0.60–0.80)	6.7% per year	8.2% per year	40 (NNTB 31– 61) per year
Primary prevention of stroke	RR 0.95 (0.85–1.06)	0.20% per year	0.21% per year	No significant benefit
Secondary prevention of stroke	RR 0.81 (0.71–0.92)	2.08% per year	2.54% per year	208 (NNTB 136– 493) per year
Major GI and extracranial bleeds in primary prevention	RR 1.54 (1.30–1.82)	0.10% per year	0.08% per year	2315 (NNTH 1525–4167) per year
Major GI and extracranial bleeds in secondary prevention	RR 2.69 (1.25– 5.76)	0.17% per year	0.07% per year	846 (NNTH 301–5715) per year
**Patients with peripheral vascular disease**				
Major cardiovacular events	RR 0.88 (0.76– 1.04)	8.9% (from 10 days to 6.7 years)	11.0% (from 10 days to 6.7 years)	No significant benefit
Nonfatal stroke	RR 0.66 (0.47–0.94)	2.1% (from 10 days to 6.7 years)	3.1% (from 10 days to 6.7 years)	95 (NNTB 61–538) (from 10 days to 6.7 years)
**Patients with diabetes**				
Major cardiovascular events	RR 0.90 (0.81– 1.00)	12.5% (from 3– 10 years)	13.7% (from 3–10 years)	No significant benefit
**Patients with pre-eclampsia**				
Risk of pre-eclampsia	RR 0.90 (0.84–0.97)	7.9% during course of pregnancy	8.7% during course of pregnancy	115 (NNTB 72–384) during course of pregnancy
**Patients with colorectal adenoma**				
Prevention of any adenoma	RR 0.83 (0.72– 0.96)	32.9% over 33 months	36.7% over 33 months	17 (NNTB 10–69) over 33 months
Prevention of advanced adenomas	RR 0.72 (0.57– 0.90)	8.7% over 33 months	11.9% over 33 months	31 (NNTB 20–85) over 33 months

### 3.1. Aspirin in cardiovascular disease

Four meta-analyses reviewed the use of aspirin therapy for cardiovascular prevention in different patient populations. One paper considered the benefits of aspirin in both primary and secondary prevention of vascular diseases [3] while the other meta-analyses reviewed the role of aspirin in other patient groups such as patients with diabetes, [56] peripheral vascular disease [57] and aspirin resistance [13].

The largest meta-analysis evaluated a variety of cardiovascular and haemorrhagic events in six primary prevention trials and 16 secondary prevention trials [3]. All the studies included in the meta-analysis were randomized controlled trials. For primary prevention, aspirin therapy was associated with a significant risk reduction in non-fatal MI (RR 0.77 95% CI 0.67–0.89), absolute risk 0.18% with aspirin *versus* 0.23% with control). However, there was no statistically significant risk reduction for important outcomes such as death from coronary heart disease (RR 0.95 95% CI 0.78–1.15, absolute risk 0.11% with aspirin *versus* 0.12% with control) or stroke (RR 1.21, 95% CI 0.84–1.74, absolute risk 0.20% with aspirin *versus* 0.21% with control). On the other hand in secondary prevention there is a significant reduction in most cardiovascular outcomes encompassing non-fatal MI (RR 0.69 95% CI 0.60-0.80), death from coronary heart disease (RR 0.87 95% CI 0.78–0.98) and stroke (RR 0.81 95% CI 0.71–0.92). The absolute risk for events in secondary prevention trial of aspirin compared to control was 6.7% *versus* 8.2% for serious vascular events, 2.08% *versus* 2.54% for total stroke and 4.3% *versus* 5.3% for coronary events. This meta-analysis also showed that aspirin therapy significantly increased major extracranial bleeds (RR 2.69 95% CI 1.25–5.76) and was associated with a trend towards more haemorrhagic strokes (RR 1.67 95% CI 0.97–2.90). In view of the potentially serious harm, the authors conclude that there is still uncertainty regarding the net value of aspirin therapy in primary prevention, and aspirin use in such patients may not be warranted due to their low absolute risk reduction with high NNTs.

Another meta-analysis reviewed the subgroup of patients with peripheral artery disease [57] and included 18 prospective randomized trials with 5,269 participants. The pooled results of any aspirin therapy compared to control group showed no statistical difference for major cardiac events (RR 0.88, 95% CI 0.76–1.04), non-fatal MI (RR 1.04 95% CI 0.78–1.39, absolute risk 3.3% with aspirin *versus* 3.6% with control), or cardiovascular death (RR 0.94 95% CI 0.74–1.19, absolute risk 4.6% with aspirin *versus* 5.2% control group). The only apparent benefit seemed to be a significant reduction in non-fatal stroke with aspirin therapy (alone or combined with other antiplatelet agents) compared to controls (RR 0.66 95% CI 0.47–0.94, absolute risk 1.8% with aspirin *versus* 3.1% with control). Treatment with aspirin monotherapy showed similar results with statistical significance for non-fatal stroke (RR 0.64, 95% CI 0.42–0.99, absolute risk 2.1% with aspirin *versus* 3.4% with control) but no significant benefit for myocardial infarction (RR 0.88 95% CI 0.36–2.14, absolute risk 3.9% with aspirin *versus* 4.5% with control), cardiovascular death (RR 1.15 95% 0.78–1.68, absolute risk 3.6% with aspirin versus 3.3% with control), and overall mortality (RR 0.96 95% CI 0.75–1.22, absolute risk 7.5% with aspirin *versus* 7.9% with control). The authors conclude that aspirin does not have significant benefit on cardiovascular events overall compared to placebo in patients with peripheral vascular disease, other than for the outcome of nonfatal stroke. This challenges the common practice of using aspirin in subgroups of patients with peripheral arterial disease who do not have any other significant cardiovascular pathology.

Cardiovascular morbidity and mortality are major concerns in patients with diabetes mellitus and a variety of treatment options have been proposed. The impact of aspirin in patients with diabetes was reviewed in a meta-analysis that included six randomized controlled trials and 10,117 participants [56]. The pooled analysis reported no significant difference between aspirin and control group for important outcomes such as myocardial infarction (RR 0.86 95% CI 0.61–1.21, absolute risk 12.5% with aspirin *versus* 13.7% with control), stroke (RR 0.83 95% CI 0.60–1.14, absolute risk 3.8% with aspirin *versus* 4.2% with control), and all cause mortality (RR 0.93 95% CI 0.82–1.05, absolute risk 11.5% with aspirin *versus* 12.3% with control). There were trends towards increased adverse effects such as any bleeding (RR 2.50 95% CI 0.76–8.21), gastrointestinal bleeding (RR 2.11 95% CI 0.64–6.95) and gastrointestinal symptoms (RR 5.09 95% CI 0.08–314.39). However this review was limited because of heterogeneity in the analysis which could not be fully explored due to limited data. The authors concluded that aspirin cannot be routinely recommended for primary prevention of cardiovascular events in patients with diabetes mellitus, and further research need to be conducted.

The fourth meta-analysis reviewed aspirin resistance and risk of cardiovascular mortality in 20 studies of 2,930 participants [13]. Only one study in the meta-analysis a prospective randomized trial and the remaining 19 were cohort studies. This meta-analysis found that there was significant increase in risk of death (OR 5.99 95% CI 2.28–15.72), acute coronary syndrome (OR 4.06 95% CI 2.96–5.56), and cerebrovascular events (OR 3.85 95% CI 3.08–4.80) among patients with laboratory defined “aspirin resistance”. This study had the advantage that 17 of the 20 trials had low risk of bias. However, amongst the limitations were the inability to evaluate the patients’ comorbidities, uncertainty as to which platelet function test is most reliable, and difficulty in ruling out treatment non-compliance as a cause of “aspirin resistance”. This study concludes that aspirin resistance adversely affects clinical outcomes but doctors should consider the evidence with caution as the overall benefit of aspirin is well proven in large trials. There is also little evidence on the best management strategies for patients who are found to be aspirin resistant on laboratory testing, nor do we know if these patients have similar or lower risks of haemorrhagic adverse effects.

### 3.2. Aspirin in obstetrics

Two meta-analyses reviewed the use of aspirin therapy in the prevention of pre-eclampsia and other outcomes of pregnancy [58,59]. One review reported the results from an individual patient data meta-analysis of 31 randomized control trials that included 32,217 women and 32,819 babies [58]. Antiplatelet agents were associated with a significant reduction of obstetric outcomes such as pre-eclampsia (RR 0.90 95% CI 0.84–0.97, absolute risk of events 7.9% with aspirin *versus* 8.7% with control) and preterm birth before 34 weeks gestation (RR 0.90 95% CI 0.83–0.98, absolute risk 6.5% with aspirin *versus* 7.2% with control) compared to placebo. However, antiplatelet use did not seem to significantly affect the risk of certain maternal outcomes such as *ante-partum* haemorrhage (RR 1.02, 95% CI 0.90–1.15, absolute risk 3.8% with aspirin *versus* 3.7% with control), Caesarian section (RR 1.03, 95% CI 0.99–1.08, absolute risk 22.9% with aspirin *versus* 22% with control) and *post-partum* haemorrhage (RR 1.06 95% CI 1.00–1.13, absolute risk 15.3% with aspirin *versus* 14.5% with control). For fetal outcomes, antiplatelet use was associated with significant reductions in preterm birth <37 weeks (RR 0.93 95% CI 0.89–0.98, absolute risk 16.8% with aspirin *versus* 18.0% with control) and need for infant ventilation (RR 0.79 95% CI 0.67–0.95, absolute risk 5.5% with aspirin *versus* 6.8% with control) but this was not significant for preterm birth <28 weeks (RR 0.87 95% CI 0.75–1.02, absolute risk 1.9% with aspirin *versus* 2.2% with control). In this meta-analysis, the authors did not find any significant benefit in reducing fetal death (RR 0.91 95% CI 0.81–1.03) or preventing growth retardation (small for gestational age) (RR 0.90 95% CI 0.85–0.96) The authors concluded that antiplatelet agents show moderate (but consistent) reductions in risk of pre-eclampsia, and preterm birth before 34 weeks’ gestation. However, there were many other outcomes where the benefits of antiplatelet therapy were unproven.

A more recent review specifically evaluated the use of aspirin in preventing pre-eclampsia and intra-uterine growth restriction in women who are at risk of pre-eclampsia [59]. This review evaluated the results of nine randomized controlled trials with 1,317 women and looked for differences in outcomes according to the time of treatment initiation. The reviewers reported that aspirin use before 16 weeks gestation was associated with a significant reduction in pre-eclampsia (RR 0.48 95% CI 0.33–0.68, absolute risk 23.3% with aspirin *versus* 47.2% with control) while treatment afterwards at 17-19 weeks (RR 0.55 95% CI 0.17–1.76, absolute risk 7.7% with aspirin *versus* 14.0% with control) and 20 weeks and beyond (RR 0.82 95% CI 0.62–1.09, absolute risk 14.3% with aspirin *versus* 17.4% with control) was not significantly better than control. Compared to control, aspirin therapy initiated before 16 weeks led to lower rates of severe preeclampsia [RR 0.38 95% CI 0.15–0.98, NNT 21 (13–50)], gestational hypertension [RR 0.61 95% CI 0.40–0.93, NNT 10 (6–50)] and intra-uterine growth restriction (RR 0.51 95% CI 0.28–0.92) but no difference in preterm birth (RR 0.93 95% CI 0.46–1.90). There was no significant difference for overall for aspirin therapy (commenced at any time) compared to control group for birth weight and gestational age at birth. This study concluded that the benefits of aspirin therapy were only observed in the subgroup of patients that were treated prior to 16 weeks gestation.

### 3.3. Aspirin in neoplastic conditions

The most recent review of aspirin therapy in preventing the development of colorectal adenomas was included in this review [60]. The meta-analysis included four randomized double-blind controlled trials that investigated the secondary prevention of colorectal adenoma in 1,289 participants in the placebo group and 1,678 participants in the aspirin (81–325 mg daily dose) group. Recurrence of adenoma was checked with colonoscopy, and the trials had a median follow-up of 33 months. This review reported that any dose of aspirin compared to placebo was associated with a reduced risk of adenoma (RR 0.83 95% CI 0.72–0.96, absolute risk 32.9% with aspirin *versus* 36.7% with control) and advanced lesions (RR 0.72 95% CI 0.57–0.90, absolute risk 8.7% with aspirin *versus* 11.9% with control). In terms of adverse events, the rate of death, myocardial infarction, major bleeding and new cancer diagnosis was not significantly different in both aspirin and placebo group. However, all reported cases of stroke occurred in the aspirin-treated group. This review concluded that trial data supports the use of aspirin in reducing the risk of recurrent colorectal adenomas.

One meta-analysis reviewed the use of aspirin therapy in chemoprotection of breast cancer [61]. This recent meta-analysis included 10 observational studies comprising of four cohort studies and six case control studies. There was a significant difference in reduction of breast cancer with aspirin therapy compared to control (RR 0.75 95% CI 0.64–0.88) in a random-effects meta-analysis. Stratifying for study design, the risk reduction with aspirin was greater in the case control studies (RR 0.70 95% CI 0.56–0.87) compare to cohort studies (RR 0.82 95% CI 0.73–0.92). Using two different models to study dose-response relationships, Berlin (ppw1 and dur1) and Il’yasova (ppw2 and dur2), both methods showed significant protective effect with high frequency of aspirin use (ppw1 = 0.002 and ppw2 = 0.001), but only the Il’yasova method was significant (dur1 = 0.06, dur2 = 0.04) for duration. The results of this review concluded that current evidence suggests the use of aspirin may reduce the risk of breast cancer and a dose-response relationship exists. However, the validity of the data is limited because of heterogeneity, difference in determining aspirin exposure, and risk of bias from the observational designs.

### 3.4. Adverse effects of aspirin in patients having surgical procedures

Two meta-analyses reviewed the haemorrhagic adverse effects of aspirin therapy in patients undergoing invasive procedures. One evaluated the use of aspirin in surgical revascularization of coronary artery disease [62] and the other reviewed the use of aspirin in cutaneous surgery [63].

One meta-analysis reviewed the risk of bleeding with pre-operative aspirin therapy [62]. This meta-analysis reviewed ten studies with 1,748 patients, with 913 patients on aspirin therapy within seven days of surgery, and 835 patients in the control (non-aspirin exposed) group. Five trials out of the ten were randomized controlled trials while the remaining five were cohort studies. Pooling of all studies showed increased chest tube loss in the aspirin group that was significantly different from the control group (WMD = 210 mL 95% CI 87–333 mL, p < 0.01). Patients in the control group has a significantly reduced requirement for blood products such as packed red blood cell transfusion (WMD = 0.65 units/patients 95% CI 0.19–1.10 units/patient, p = 0.01) and fresh frozen plasma (WMD = 0.61 units/patient 95% CI 0.07–1.16 units/patient, p = 0.03), with a trend towards for platelet infusion (WMD = 0.6 units/patient 95% CI 0.04–1.24 units/patient, p = 0.06). Reexploration for bleeding was increased in the group treated with aspirin which was significant (RR 2.32 95% CI 1.31–4.08, p < 0.01). This review concluded that patients who receive aspirin within seven days of surgery were at higher risk of blood loss, but the validity of the data was limited because many studies were of low methodological quality.

Aspirin use in dermatological surgery is associated with increased risk of post operative bleeding but consensus about anticoagulation use is less clear. Studies have reported mixed results regarding the risk of postoperative bleeding and complications [64,65,66,67]. A meta-analysis of four prospective studies and two retrospective studies with a total of 1,373 patients reported that aspirin or NSAIDs was associated with increased risk of moderate to severe complications compared to controls (OR 2.0 95% CI, 0.97–4.13, p = 0.06). This study concluded that there is a low but significant risk of complications associated with use of anticoagulants but this risk is greater with warfarin than aspirin.

### 3.5. Aspirin and Gastrointestinal Harm

The effects of aspirin on gastrointestinal haemorrhage were systematically reviewed by Derry and Loke almost 10 years ago [47]. This meta-analysis found a NNT for harm of 106 over 28 months, with a fairly flat dose-response curve over the range 50 mg–325 mg, and little evidence for the purported benefits of modified release formulations. A more recent ‘best evidence’ review focusing on enteric-coated aspirin concluded that the available data showed no significant risk reduction for gastrointestinal bleeding or ulceration. [68]. Serious complications from upper gastrointestinal bleeding and perforation continues to be a major problem, with a recent systematic review showing that since 1997, mortality rates from gastrointestinal complications have continued to rise in aspirin and non-steroidal anti-inflammatory users [69].

## 4. Conclusions

This overview of recently published meta-analyses suggests that aspirin therapy has significant benefits in a variety of clinical settings, but there are still uncertainties that will require more research. Current evidence suggests that aspirin is beneficial for secondary prevention for cardiovascular disease, primary prevention of pre-eclampsia, and secondary prevention of colorectal adenomas. However, use of aspirin in primary prevention of cardiovascular disease, or in patients with diabetes mellitus, or in those with peripheral vascular disease is not supported by the current evidence. It is also clear that aspirin has considerable potential for harm in patients undergoing surgical procedures. The benefits of aspirin use must be weighed clinically against the risk of adverse events such as gastrointestinal and intracranial bleeding. 
